# Supplementary material for: Designing a Cross-Cultural Bridging Intervention to Increase Under-Served Immigrant Parents’ Engagement in Evidence-Based Online Parenting Programs: A Co-Design Study with Indian-Origin Parents in Australia
Source: Children (Basel). 2025 Aug 30;12(9):1158. doi: 10.3390/children12091158 (PMC12468362; doi:10.3390/children12091158)

Designing a Cross-Cultural Bridging Intervention to Increase Under-Served Immigrant Parents' Engagement in Evidence-Based Online Parenting Programs: A Co-Design Study with Indian-Origin Parents in Australia

Sunita Bayyavarapu Bapuji [1,*], Ling Wu [2], Joshua Seguin [2], Patrick Olivier [2], and Marie Bee Hui Yap [1,*]

Supplementary file

Description of three sets of workshops

The first set of three workshops (W1n = 4, W2n = 3, and W3n = 5 participants) explored participants' knowledge about parenting programs and the barriers to engaging in them. In these workshops, participants were first made aware of growing concerns about youth mental health problems and were presented with insights from the earlier two studies pertaining to Indian immigrant families (See example scenario in Figure 1). After providing the background information, the facilitator invited Indian parents to role-play as an advisory committee (See example in Figure 2), discussing the barriers to engaging with freely available parenting programs while adding their discussion points on the whiteboard and subsequently facilitating a discussion of strategies to increase Indian-origin parents' awareness and engagement in parenting programs. Figure 3 shows an example of different participant perceptions and experiences about barriers to engaging in parenting programs.

The second set of two workshops (W4n = 6, W5n=4, and W6n = 2 participants) used vignettes (see Figure 4 for an example) to prompt reflections and discussions of their own or other known Indian immigrant parents' lived experiences. Participants discussed strategies and their prioritisation for inclusion in the bridging intervention to empower and support immigrant families facing parent-child acculturation concerns to maintain/support youth well-being. The third set of three workshops (W7n = 2 and W8n = 2 participants) focused on sense-checking the findings from the previous two sets of workshops and facilitating discussion to further prioritise strategies for inclusion in the bridging intervention, which is intended to support and increase Indian immigrant families' engagement in existing parenting programs. The same vignettes presented in the second set of workshops were used for sense-checking in the third set of workshops. Finally, a design ideation workshop with the research team (n = 4) was conducted to ideate design principles for the bridging intervention, to bridge between Indian-origin parents and existing parenting programs.

Figure S1. Scenario used in the first set of workshops to explain the acculturation conflicts

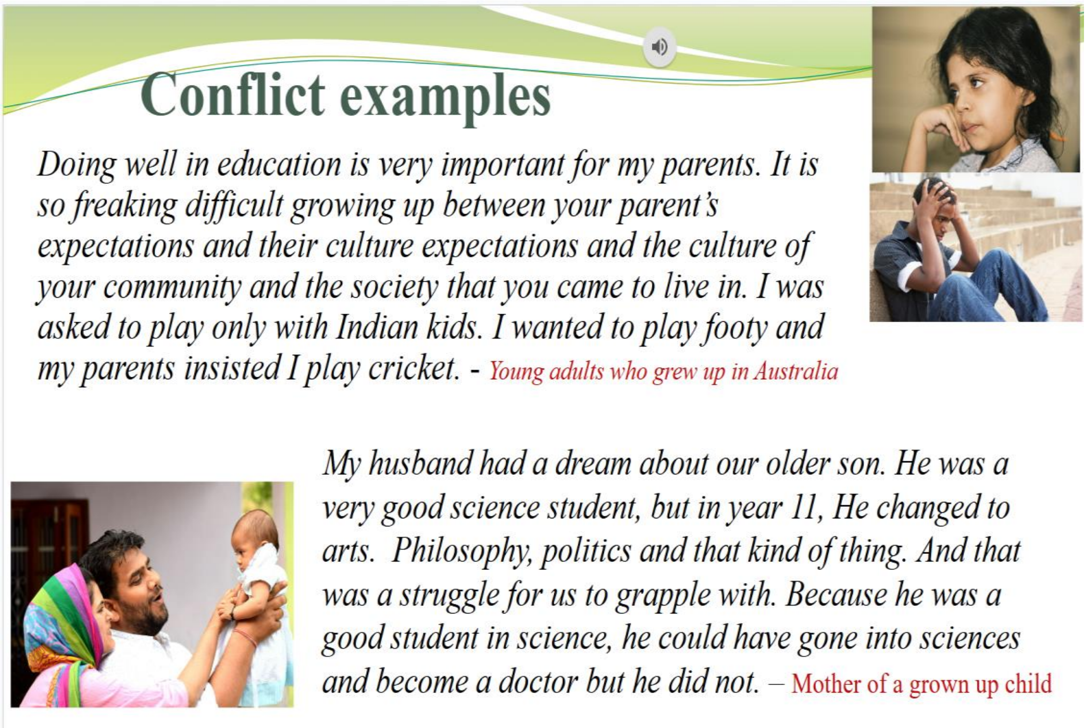

Figure S2: Role-play slide used in the first set of workshops

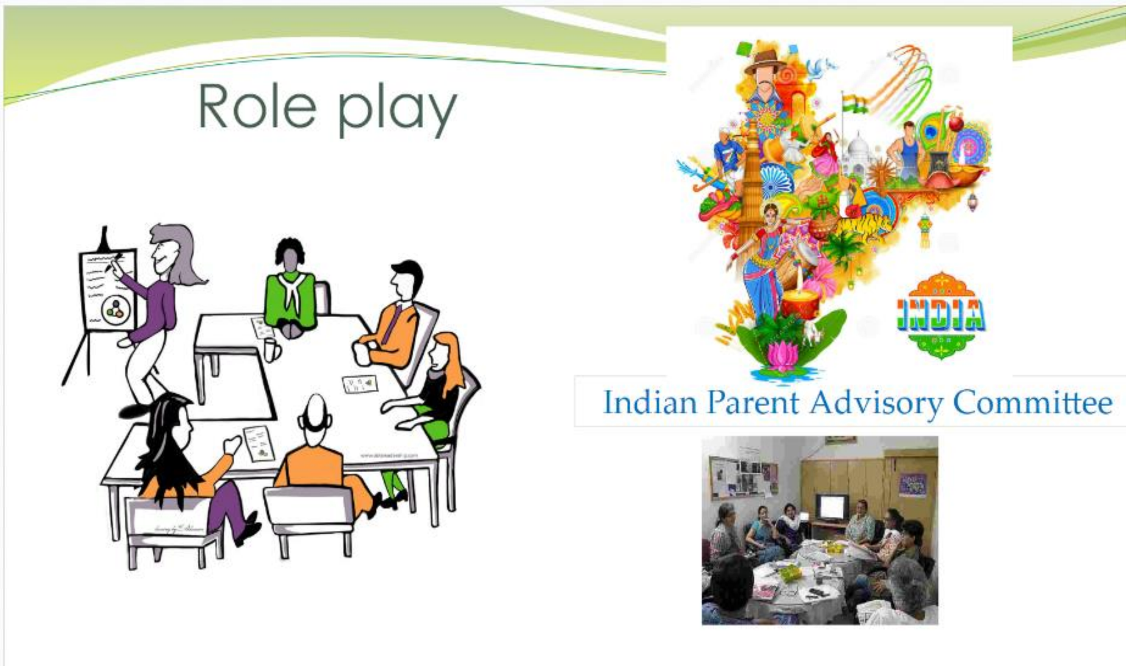

Figure S3: An example of role-play discussion notes captured on the whiteboard

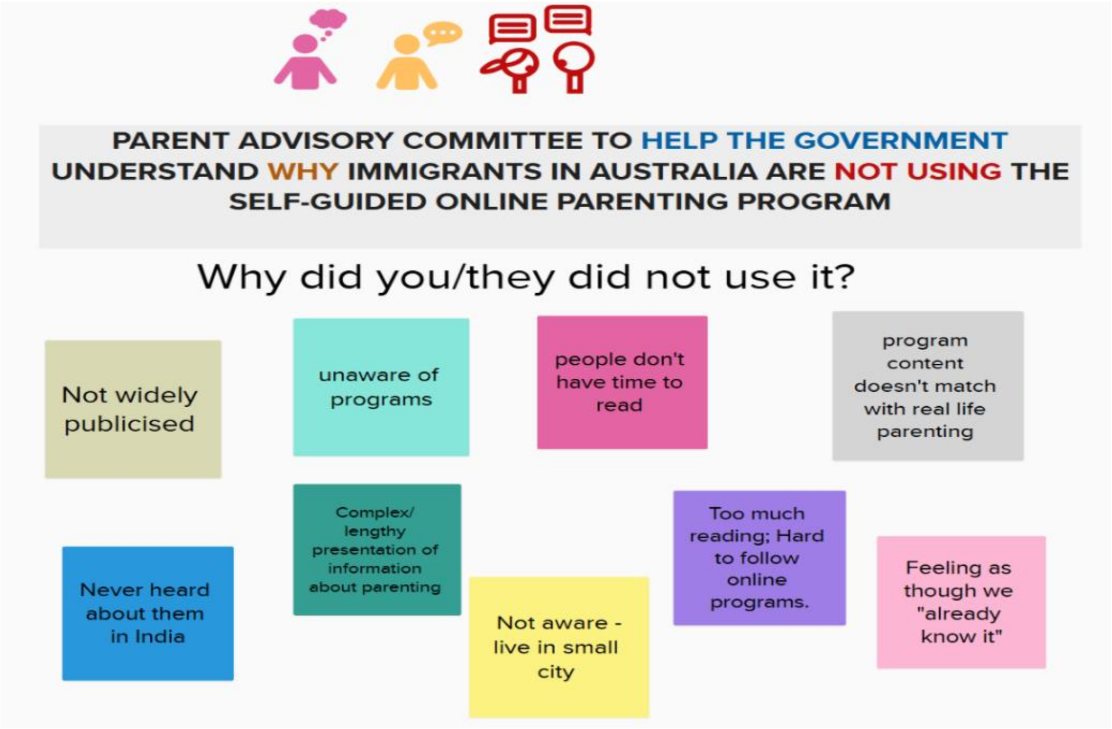

Figure S4: Vignette used in the second and third sets of workshops

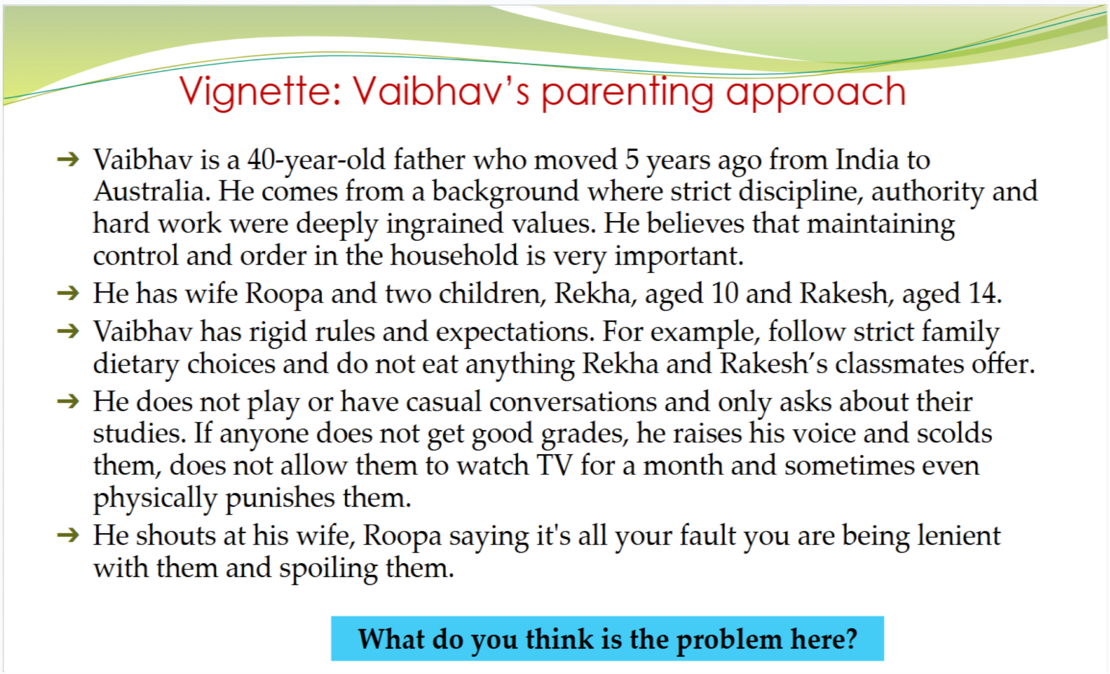

Supplement: Supplementary file 1 [file children-12-01158-s001.zip › children-3811444-supplementary.pdf]
